# Continuity care in surgery: our experience

**DOI:** 10.1186/1471-2318-11-S1-A16

**Published:** 2011-08-24

**Authors:** G Ferrocci, A Marzetti, C Rossi, L Possamai, E Durante, G Azzena

**Affiliations:** 1 Istituto di Chirurgia Generale, Azienda Ospedaliero-Universitaria S.Anna, Università di Ferrara, Ferrara, Italy

## Background

For many years some demographic changes have been taking place that means also a constant growth of the elderly population. These changes cause an increase of “frail” patients, affected by chronic and degenerative diseases, often associated and affected by functional limitations and/or disabilities [1]. That has lead to an increasing demand for long term social and medical services with a subsequent rise in sanitary aids consumption. The traditional model of care offering specialist and episodic treatments, appears to be no longer suitable to guarantee the best results regarding a good quality of life and abilities [2].

## Materials and methods

Patients arriving to our Operative Unit represent a selected group, affected by different severe diseases, with the association of high disability and difficult social status, that often leads to admission to Institutes and to higher incidence of mortality (that is weakly elderly persons)[3,4].

From January 2008 to October 2010 we hospitalized 2074 elderly patients (1275 men, 799 women). The average age was 75.5 (from 65 years to 108 years).

Patients admitted to long-term Institutes from our Unit are 185 (8.9%).

## Results

In surgery, the elderly patient can complicate not only the surgical operation but also the post-operative period, because of many associated diseases becoming acute.

At the end of the hospital stay, these patients could still need medical assistance, nursing and/or rehabilitation organized in an integrated project of variable length [5]. This integrated model is based on a net system of a series of flexible structures and services that offer the answer to the assistance needs of the elderly person: integrated home assistance, sanitary residence assistance, hospitalization at home, long-term stays after disease and geriatric unit for acute patients. The main purpose is not the recovery, but the functional rehabilitation and the improvement of the quality of life of the patient [6].

## Conclusions

The real geriatric patient is the frail elderly person with a higher risk of developing disabilities and consequently the loss of self-sufficiency. Problems come out especially at the end of a hospital stay, that is the critical end point for the elderly person [1]. The need to extend the time of treatments and sanitary care lead to the creation of a “model of cronic care”. Thus the purpose became to “take care”, through a global approach to improving the quality of life of the patient and to reduce the incidence of disability.
